# Mating Plugs in Polyandrous Giants: Which Sex Produces Them, When, How and Why?

**DOI:** 10.1371/journal.pone.0040939

**Published:** 2012-07-19

**Authors:** Matjaž Kuntner, Matjaž Gregorič, Shichang Zhang, Simona Kralj-Fišer, Daiqin Li

**Affiliations:** 1 Institute of Biology, Scientific Research Centre, Slovenian Academy of Sciences and Arts, Ljubljana, Slovenia; 2 Department of Entomology, National Museum of Natural History, Smithsonian Institution, Washington, D.C., United States of America; 3 College of Life Sciences, Hubei University, Wuhan, Hubei, China; 4 Department of Biological Sciences, National University of Singapore, Singapore, Singapore; National Cancer Institute, United States of America

## Abstract

**Background:**

Males usually produce mating plugs to reduce sperm competition. However, females can conceivably also produce mating plugs in order to prevent unwanted, superfluous and energetically costly matings. In spiders–appropriate models for testing plugging biology hypotheses–mating plugs may consist of male genital parts and/or of amorphous covers consisting of glandular or sperm secretions. In the giant wood spider *Nephila pilipes*, a highly sexually dimorphic and polygamous species, males are known to produce ineffective embolic plugs through genital damage, but nothing is known about the origin and function of additional conspicuous amorphous plugs (AP) covering female genitals.

**Methodology:**

We tested alternative hypotheses of the nature and function of AP in *N. pilipes* by staging mating trials with varying degrees of polyandry. No APs were ever formed during mating trials, which rules out the possibility of male AP formation. Instead, those females that oviposited produced the AP from a liquid secreted during egg sac formation. Polyandrous females were more likely to lay eggs and to produce the AP, as were those that mated longer and with more total insertions. Our further tests revealed that, in spite of being a side product of egg sac production, AP, when hardened, prevented any subsequent copulation.

**Conclusions:**

We conclude that in the giant wood spider (*Nephila pilipes*), the amorphous mating plugs are not produced by the males, that repeated copulations (most likely polyandrous) are necessary for egg fertilization and AP formation, and that the AP represents a female adaptation to sexual conflict through prevention of unwanted, excessive copulations. Considering the largely unknown origin of amorphous plugs in spiders, we predict that a similar pattern might be detected in other clades, which would help elucidate the evolutionary interplay of various selection pressures responsible for the origin and maintenance of mating plugs.

## Introduction

The phenomenon of plugged female genitalia is common in several animal groups. Among vertebrates, copulatory plugs of various forms and functions are known in diverse taxa of mammals [1]–[6], and reptiles [7], [8]. There is, however, much more variety in the form, function, and origin of mating plugs in invertebrates, known in e.g. nematodes [9], [10], acanthocephalan parasites [11], crustaceans [12], [13], insects [14]–[21], and arachnids [22]–[24]. Although mating plugs are relatively widespread across animal taxa and their function is well studied, little is known about their proximate mechanisms.

The vast majority of documented invertebrate mating plugs are produced by males either through glandular secretions [15]–[27] or ejaculates [13]–[31], or by utilizing severed male somatic [23] or genital parts [32] as copulatory barriers. The latter phenomenon, termed ‘mate plugging through genital mutilation’ [33], [34], or simply ‘emasculation’ [35], has been shown to serve male’s paternity protection [35]. Male initiated plugging is an adaptation to sperm competition [36], because plugged females are monopolized and are thus unavailable to subsequent males as long as plugs remain effective [16], [20], [23], [27], [35]. Additionally, plug substances may lower female receptivity [37], [38] or female attraction [39], or perhaps even prevent sperm dumping [40]. Very rarely, however, are genital plugs produced by female secretions in addition to male secretions [41] and these may be seen as serving to prevent superfluous, unwanted matings. Thus, although both male and female produced plugs function analogously through prevention of subsequent copulations, they must arise through entirely different, even sexually conflicted, mechanisms.

A game theory model of mate plugging only predicts male initiated plugs [42]. In spiders, it seems that the vast majority of plugs are indeed male produced while female plug (co)production is rare [24], [41], [43]. The best documented are those spider plugs that arise through male genital mutilation [32]–[36], [44]–[48], and several studies also document amorphous plugs consisting of male glandular or sperm secretions [24], [27]. In fact, the literature is nearly devoid of any evidence of female produced plugs in spiders. Notable exceptions are a handful of taxa where the females help the males in amorphous mating plug formation. For example, in the tetragnathid spider *Leucauge mariana*, male courtship patterns elicit female cooperation in plug formation [43], [49], and in *L. argyra*, females produce a whitish liquid substance during copulation, which solidifies into a genital cover [50]. Furthermore, in *Theridion varians*, the mating plug consists of both male and female secretions from their respective genital tracts [41]. However, female spiders are rarely reported to be entirely in charge of mating plug formation [51], [52]. A recent review established 206 spider species known for mating plugs [24]. Of these, 141 (68%) are reported to be made of amorphous secretions, while 61 (30%) consist of male genital parts, and only in four cases (2%) amorphous and male parts are combined. However, we are only beginning to understand the patterns: in the majority of taxa with amorphous plugs (116 cases) the sex which produces them is unknown, while in 22 cases it is the males that produce them, and in only three cases it is the females, and in a single case, both sexes together [24]. We are thus facing a largely unknown origin of amorphous mating plugs in spiders, with the correspondingly spurious understanding of spider mating plug biology.

**Figure 1:**
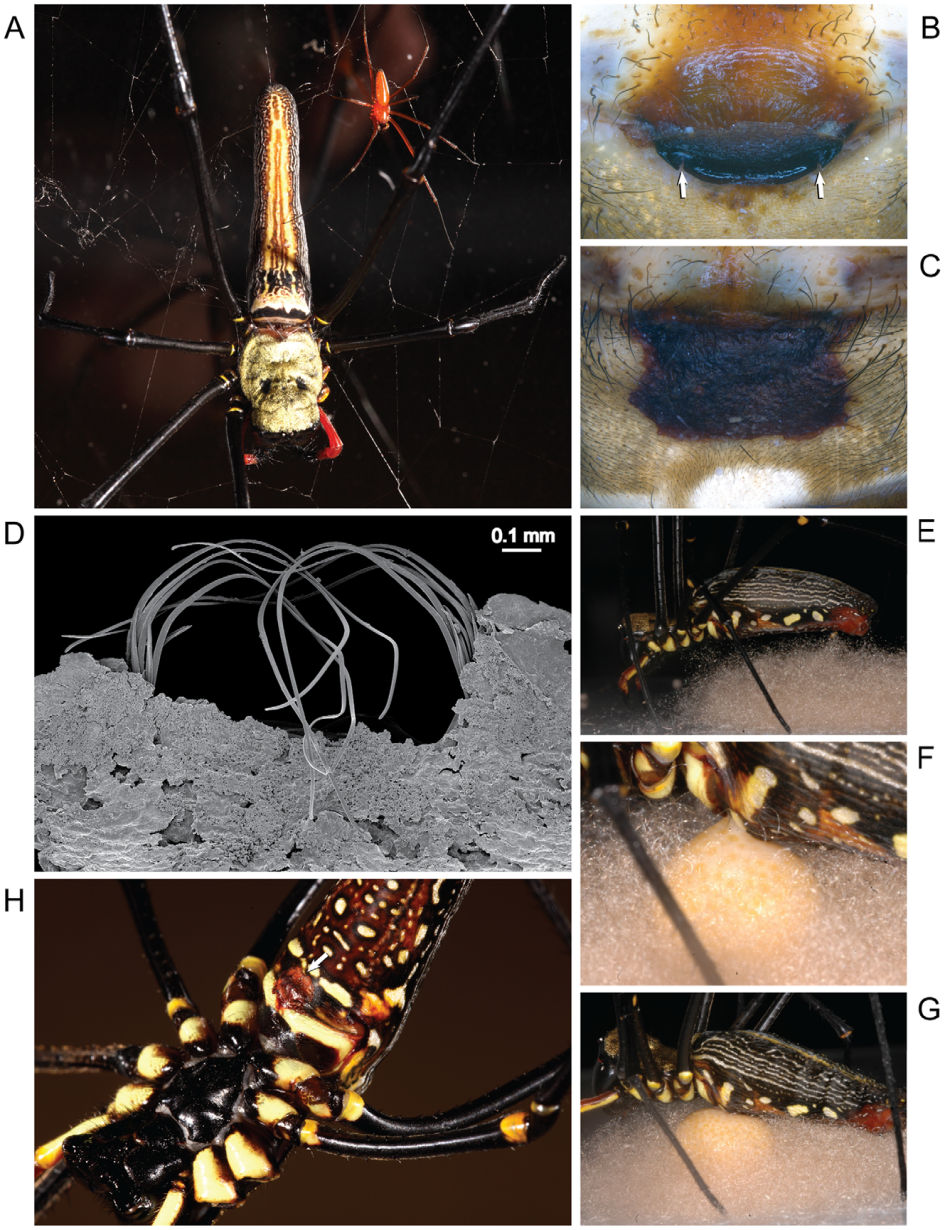
The amorphous plug formation in the giant wood spider *Nephila pilipes*, a highly polygamous and sexually dimorphic species, takes place not during mating, but during oviposition. A, giant female (left) and small male (right); B, female genital area, intact, arrows indicate paired copulatory openings; C, female genital area covered with a hard amorphous plug (AP); D, two types of plugs in a female, image shows a removed AP with several male embolic leftovers lodged in each copulatory opening; E–G, female spins an egg sac (E) and deposits in it fertilized eggs (F–G); the red AP covering the epigynum (H, arrow), is thus a side product of egg-laying, yet it functions as copulation prevention (see text). Images by M. Kuntner (A, D, H), M. Gregorič (B, C) and S. Zhang (E–G).

Spiders are highly suitable organisms for sexual biology research [53], [54], and the family Nephilidae contains particularly good taxonomic models for studying mating plug biology and its implications for sexual selection [45], [55], [56]. Within nephilids, male and female genitalia apparently coevolved from simple to complex and back to simple in a unique display of evolutionary arms race [57], where more complex male genital plugs enforce female monandry (e.g. *Herennia*, *Nephilengys*). Through simplification of female and male genitals, however, the male produced mating plugs became ineffective, and thus the females of phylogenetically derived *Nephila* were able to reassert polyandry [45]. The most dramatic case of the documented polyandry comes from the giant wood spider *Nephila pilipes*, a highly sexually dimorphic species (Fig. 1A), where females commonly sport multiple embolic plugs [45]. In addition to these male-produced embolic plugs found in female copulatory openings, female *N. pilipes*, uniquely among nephilids, also commonly possess a conspicuous, reddish amorphous plug (hereafter AP), which covers the entire epigynal area (Fig. 1B–D,H) [45]. Presumably, such hardened AP blocks additional male access to female copulatory openings. However, such AP function has not been tested empirically, and it remains unknown even which sex produces it, when, and how [45].

By staging mating trials with varying degrees of polyandry, we addressed three salient questions about the AP phenomenon in *N. pilipes*: 1. Which sex produces the AP (male, female or both) and how is it formed? 2. When in relation to mating occurrence is the AP produced? and 3. Does the AP prevent copulation? Considering the fact that male *N. pilipes* commonly produce embolic plugs [45], it is most likely that males also produce the AP. If this hypothesis proved valid, we predicted to observe male induced AP formation, by means of documenting secretion during mating. Similarly, if cooperation of both sexes was the mechanism for AP production, it should also take place during the presence of the male. Solely female produced plugs could only be definitively determined if their observed production was subsequent to mating in the absence of any male. If so, we predicted to document female AP production only after several matings and shortly before or during oviposition. This is because female produced plugs should function as protection against unwanted male harassment. Based on this logic, we predicted that multiply mated females and older females would be more likely to produce the plugs, and to produce egg-sacs. Finally, in order to term these structures ‘mating plugs’, we tested their function, which presumably is the prevention of superfluous copulations. We thus staged additional mating trials of intact males with (naturally) plugged females.

## Results

All matings observed followed the general patterns already described in the literature [45], [58], [59], whereby the males do not follow particular courtship rituals prior to first copulations, but rather approach the female directly on her venter to copulate (Video S1, S2), then continue to ride the giant female to perform ‘mate binding’ in-between copulations [60] (Video S3). Subsequent to trials, we found male genital leftovers (embolic plugs; Fig. 1D) in the spermathecae of four out of 16 (25%) females (Table 1).

**Table 1:** Summary data from the control (CG) and three experimental groups (EG): females in EG1, 2 and 3 mated with one, three, and five males, respectively.

Group	Female code	Female weight(g)	Total no. of insertions	Total copulationduration (s)	Embolic plugs (L/R)	Plug during trial	Egg laying	Amorphous plug	Adult molts	Post egg-laying molts	Genitalia molted
CG	F012	0.44	0	0	0/0	–	n	n	1	–	–
CG	F013	1.07	0	0	0/0	–	n	n	1	–	–
CG	F014	0.57	0	0	0/0	–	n	n	1	–	n
CG	F023	0.60	0	0	0/0	–	n	n	1	–	n
CG	F070	–	0	0	0/0	–	n	n	0	–	–
CG	F093	0.40	0	0	0/0	–	n	n	1	–	n
CG	F101	1.15	0	0	0/0	–	n	n	1	–	n
CG	F106	–	0	0	0/0	–	n	n	1	–	–
CG	F107	0.43	0	0	0/0	–	n	n	2	–	n, –
CG	F108	–	0	0	0/0	–	n	n	0	–	–
EG1	F041	–	1	509	–	n	n	n	1	–	–
EG1	F043	–	1	2009	–	n	n	n	1	–	–
EG1	F046	0.64	7	335	1/0	n	n	n	1	–	–
EG1	F057	1.32	1	4668	0/0	n	n	n	1	–	n
EG1	F061	0.40	1	4135	0/0	n	n	n	0	–	–
EG1	F084	1.13	1	3202	1/1	n	n	n	1	–	–
EG1	F091	–	2	3556	–	N	n	n	0	–	–
EG1	F094	0.85	1	2039	0/0	n	n	n	1	–	n
EG1	F096	1.16	4	1749	0/0	n	n	n	1	–	–
EG2	F003	1.11	12	2576	0/1	n	y	y	1	n	n
EG2	F032	0.74	8	6622	0/0	n	n	n	1	–	–
EG2	F033	1.20	5	4943	0/0	n	y	y	1	n	n
EG2	F039	1.64	7	4758	0/0	n	n	n	1	–	n
EG2	F042	1.21	7	5724	0/0	n	n	n	1	–	n
EG2	F055	0.67	9	1887	0/1	n	y	y	1	n	n
EG2	F060	0.66	6	1340	0/0	n	n	n	2	–	n; n
EG2	F066	–	6	3804	–	n	n	n	0	–	–
EG2	F082	–	5	2662	–	n	n	n	0	–	–
EG2	F095	0.77	13	7837	–	n	n	n	1	–	–
EG3	F001	1.42	9	10507	0/0	n	n	n	0	–	–
EG3	F004	1.18	12	7193	0/0	n	y	y	0	n	–
EG3	F015	–	6	6504	–	n	n	n	0	–	–
EG3	F018	1.03	12	3964	0/0	n	y	y	1	n	–
EG3	F021	–	20	5421	–	n	n	n	0	–	–
EG3	F022	–	19	6312	–	n	y	-	0	n	–
EG3	F036	0.65	15	5612	–	n	y	y	2	n	n, -
EG3	F044	1.06	28	8389	–	n	n	n	0	–	–
EG3	F053	0.38	10	7738	–	n	y	y	1	n	–
EG3	F075	1.76	15	10467	–	n	y	y	2	n	n; n

L = left, R = right, n = no, y = yes, -  = inapplicable.

**Figure 2:**
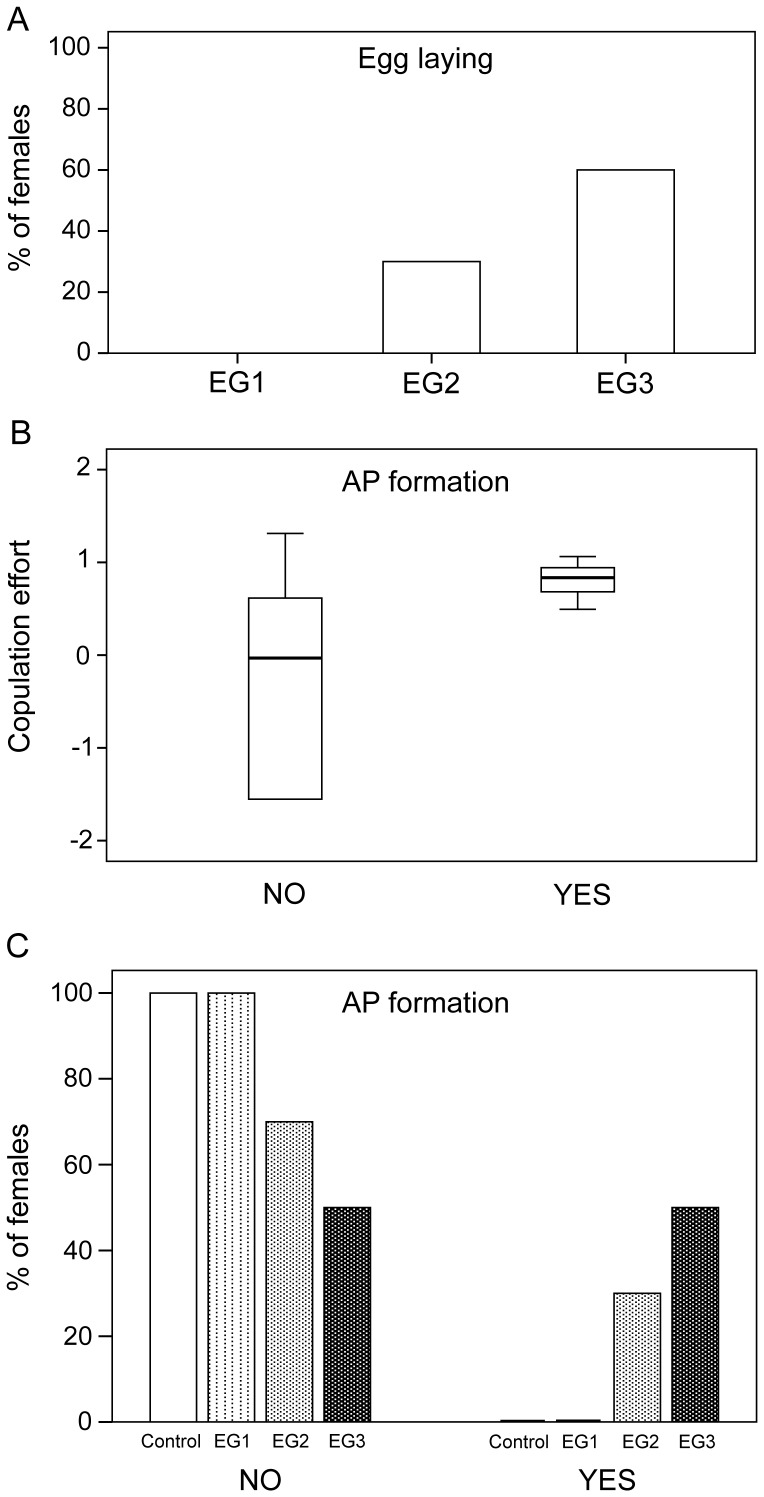
Exploration of oviposition, copulation effort and amorphous plug (AP) formation. A, share of egg-laying females per experimental group (EG1 = 1 male, EG2 = 3 males, EG3 = 5 males); B, copulation effort (number of insertions and copulation duration combined) in females with and without AP; C, share of females per EG with and without AP.

No APs were ever formed during mating (Table 1), which rules out the possibility of male plug formation. Nine out of 29 females (31%) produced eggs. No female from EG 1 oviposited, while three females from EG 2 (30%) and six from EG 3 (60%) produced an egg-sac (Fig. 2A), and of these, two females from EG3 also produced a second egg-sac. A typical oviposition repertoire involved the female first spinning a layer of silk as the basis for the egg-sac (Fig. 1E), then releasing the egg mass from the uterus externus (Fig. 1F) with the egg mass forming a sphere within the single egg-sac (Fig. 1G), then covering it with another layer of silk. Subsequent monitoring revealed spiderlings’ hatching from these egg-sacs. The females that produced an egg-sac also produced the epigynal AP during the first egg-laying. The AP consisted of a liquid red secretion that was excreted during egg-sac formation, and later hardened (Fig. 1H).

The logistic regression showed that egg-sac laying strongly co-occurred with AP formation, and both depended on copulation effort (Table 2A–B; Fig. 2B). AP formation also depended on the number of males with which a female copulated, although not significantly (Table 2A; Fig. 2C). Probabilities for egg-sac laying increased with the number of males and with copulation effort (Table 2B).

**Table 2:** Results from conditional backward logistic regressions testing factors that significantly affected (a) occurrence of amorphous plug (AP, independent variables: number of males with which a female copulated (experimental group; EG), egg-laying and copulation effort); (b) occurrence of egg-laying (independent variables: number of males with which a female copulated (EG), AP formation and copulation effort); and (c) molting in adulthood (independent variables: number of males with which a female copulated (EG), AP formation, egg laying, copulation effort and mass).

(a)					
Variable		Model Log Likelihood	Change in -2 Log Likelihood	df	Sig. of the change
Step 1	Egg laying	−64.472	128.944	1	<0.001
	EG (no. of males)	−3.806	7.611	3	0.055
	Copulation effort	−8.376	16.752	1	<0.001
**(b)**					
**Variable**		**Model Log Likelihood**	**Change in -2 Log Likelihood**	**df**	**Sig. of the change**
Step 2	Copulation effort	−6.662	7.908	1	0.005
	AP	−27.639	49.862	1	<0.001
**(c)**					
**Variable**		**Model Log Likelihood**	**Change in -2 Log Likelihood**	**df**	**Sig. of the change**
Step 3	EG (no. of males)	−11.646	12.882	3	0.005
	AP	−8.424	6.438	1	0.011

The test of the function of the AP through remating experiments (N = 6) showed that none of the intact males succeeded in copulating with naturally plugged females (N = 4). These results thus indicate that the function of the AP is copulation prevention.

27 of 39 females (69%) molted after maturity, including those from the control group. As we report elsewhere, these molts did not contain any genital structures, which suggested that the molted females retained their inner genitals (spermathecae and ducts) and the stored sperm. No adult female molted after egg-sac and plug formation. The probability of post maturity molting decreased with the number of males with which the female copulated and with the presence of AP (Table 2C).

No statistically significant difference was found in the longevity of adult females from different groups (control: 92±39 d, EG1∶106±26 d, EG2∶125±41 d, EG3∶110±51 d; Kruskal-Wallis χ^2^ = 5.35, df = 3, p = 0.148).

## Discussion

We examined the nature of amorphous mating plug formation in *N. pilipes*, and its function as remating prevention. We found that the plug is not made by the male, but is rather formed by the female as a side product of oviposition. We also confirmed the prediction that this amorphous plug (AP) functions as copulation prevention. We thus interpret the AP in *N. pilipes* as a female adaptation to control unnecessary male copulation attempts. Our results further suggest that a substantial mating effort is necessary for the female to secure enough sperm for oviposition. During this research we also found that *N. pilipes* females continue to grow and molt after maturity, which seems to be unique among orb weavers, and has implications for the understanding of developmental mechanisms underlying female gigantism.

The AP of *N. pilipes* covers the entire female genital area and thus differs from other types of plugs found in *Nephila* species and related spiders, where plugs are parts of males’ intromittent organs [35], [45], [48], [57]. There, plugging through genital emasculation with the intent to prevent subsequent male copulation is a male adaptation to sperm competition [57]. In fact, plugging through male genital damage also takes place in *N. pilipes* (Table 1); however, while such embolic plugs seem to be fully efficient in certain nephilid spiders [35], [45], they fail to prevent subsequent matings in *N. pilipes* [45]. From this end, the possibility that the males might have evolved an additional mechanism through amorphous plugging of the female genital area seemed to be plausible. However, our study discarded this possibility in favor of the alternative. Thus, it is the female that produces the AP during oviposition.

A mating plug theoretical model [42] builds on the assumptions that males are the sex that plugs, and that the plugs can be removed by subsequent males. Although the model allows for the possibility that females might benefit from being plugged through reduced harassment rates or that they even assist in plug formation [42], it does not foresee solely female produced plugs. Several studies have investigated amorphous plugging in spiders, and they predominantly found that these are formed through male secretions [24]. For example, *Amaurobius* males produce APs using secretion from a gland that opens at the embolus base [61], and *Agelena* males produce mating plugs that enhance the first male’s fertilization success [27]. In some cases, for example in *Theridion*, plugs are produced by a combination of male and female secretions [41], and in *Leucauge mariana*, females may assist in male plug formation depending on his courtship behavior [49]. However, in *N. pilipes*, males do engage in embolic, but ineffective plugging [45], but it is the females that produce the amorphous, and effective plugs themselves. Considering the largely unknown plug origin in the majority of those spider species known for APs [24], we find it likely that subsequent research will find many more cases of female plug production, the evolution of which the existing models cannot elucidate. It would be worthwhile to reexamine the reports from the older literature that in some *Agelena* species mating plugs are also female produced ([51]–[52] cited in [61]).

In our study, none of the females that mated only once oviposited, while 30% and 60% of those that had mated with three and five males, respectively, produced viable egg-sacs (Fig. 2A). In fact, those females that copulated with more males, for a longer total copulation time, and a higher total insertion number, were more likely to lay eggs (Fig. 2B–C). This suggests that a single copulation is not sufficient for *N. pilipes* females to fertilize eggs. The trend in our data suggests that oviposition would be closer to 100% only if females were to mate with more than five males. However, perhaps the same may be achieved through a single male continuously mating the female for several days, as we had previously observed [45] and is also known in other *Nephila* species [62]. With the current data we cannot unequivocally distinguish between the need for several copulations with the same male versus with different males. However, being that *N. pilipes* females are known to be extremely polyandrous [45], we lean towards the interpretation that polyandry is in fact necessary for successful egg fertilization.

Although the number of our remating experiments that involved naturally plugged females was only six, this simple test of the AP function revealed that no males were able to copulate with plugged females. It thus seems that, although technically the AP is a by-product of oviposition, such plug indeed functions as a copulation barrier once it hardens. It is difficult to explain why females produce such a plug. We argue that while it is in the female interest to be polyandrous, it is not in her interest to be a recipient of excessive matings after oviposition [9]. The AP thus enables females to prevent superfluous copulations, which otherwise impose an excessive energetic cost to the female without a significant benefit.

In our laboratory tests, only two females produced more than a single egg-sac, but the second one was never viable. Although these data might suggest that *N. pilipes* females mostly produce a single egg-sac in their lifetime, the situation in nature is clearly different, as prior studies report no less than 89 egg sacs having been produced by 10 females in a year in Papua New Guinea [58]. Because our data show that all females produce an AP during first egg-laying, this would either imply that further egg-laying is possible after AP has formed, or that the females possess the ability to remove it for further oviposition, or even for further mating. More research is needed to investigate whether females are able to oviposit even in the presence of AP, and if so, what the mechanism is.

We found that the females that copulated with fewer males and had not produced an egg-sac were more likely to continue adult growth through post-maturity molting. The female mass however, seems to play no role in post-maturity molting. This suggests that regardless of her size, the female’s interest seems to be continuous growth until she has accumulated enough sperm for egg fertilization through repeated polyandrous copulations. While the females benefit from continuous growing and molting, likely responding to fecundity selection [63], they do not molt any cuticle associated with inner genitals [64]. Logically, the spermathecae are essential for growing females to retain, as they might already contain sperm. After oviposition, however, no further molts were observed, and the females simply persisted, plugged, until they died.

### Conclusions

The currently understood mating biology of the giant wood spider *N. pilipes* encompasses a plethora of behavioral adaptations arising through sexual selection [45], [60], among which are both male- and female-produced mating plugs. We conclude that 1. the previously unstudied amorphous mating plugs represent a female adaptation to sexual conflict through prevention of unwanted, excessive copulations, and that 2. repeated copulations or polyandry are necessary for egg fertilization and AP formation. Considering the largely unknown origin of amorphous plugs in spiders, we predict that a similar pattern of female produced amorphous mating plugs will also be discovered in other spider clades, which would help elucidate the evolutionary interplay of various selection pressures responsible for the origin and maintenance of mating plugs.

## Materials and Methods

We collected adult males and subadult females of *N. pilipes* on Pulau Ubin, Singapore (N 1.421575°, E 103.932542°). To control for female virginity, we placed 40 subadult females into individual 50 cm×50 cm×10 cm perspex frames and reared them to adulthood. We placed 155 adult males with intact palps into individual 100 ml plastic cups. Additionally, we collected four adult females naturally sporting AP to test for plug effectiveness by means of preventing subsequent copulations. We watered all spiders daily. Three times a week, we fed the females with flies and mealworm larvae and the males with fruit flies.

We grouped the virgin females into a control and three experimental groups. In the control group (CG, N = 10) the females were not exposed to any males. Females from the experimental groups were subjected to mating trials, where we introduced one randomly selected male to the female for an hour, during which we observed mating behavior, i.e. courtship, number and duration of insertions in each copulatory opening, and noted potential plug formation. If a male never inserted his palp for more than two seconds, we considered the copulation as failed, because at such short insertion attempts the males failed to expand their palpal haematodochae, which is necessary for sperm transfer [53]. In the experimental groups (EG), we assigned females to varying degrees of polyandry: In experimental group 1 (EG1, N = 9) each female mated with a single male, in experimental group 2 (EG2, N = 10) each female mated with three different males, and in experimental group 3 (EG3, N = 10) each female mated with five different males. No males were used more than once in trials. After all trials we allowed the females at least a full day rest before presenting those in the polyandrous trials (EG2, EG3) with another male.

In order to test for plug effectiveness, we introduced intact males to the four naturally plugged females for a total of six mating attempts and followed the protocol of the experimental groups.

We video recorded haphazardly selected trials using Canon SLR cameras in order to document the main behavioral repertoires (Video S1, S2, S3). After the trials, we weighted the males to the nearest 0.01 mg, and then preserved them in ethanol. We reared and monitored the females and examined them for plug formation daily for the remainder of their lifespan. Because some of these adult females continued to molt after maturity, we preserved their exuviae for subsequent inspection of whether any genital parts were also molted. Upon the end of all trials we weighted the females to the nearest 0.01 mg and preserved them in ethanol. We then examined the genitalia of all females for the number of embolic plugs by dissecting their spermathecae, following previously established protocols [45].

We analyzed the factors influencing egg-sac laying and AP formation (dependent variables) using conditional backward logistic regression. Independent variables included the number of males with which a female copulated (EG group), AP formation (when egg-sac laying was used as the dependent variable) or egg-sac laying (when AP formation was used as the dependent variable), respectively, and copulation effort. Copulation effort was estimated with regression score combining the total number of insertions per female and total copulation duration per female calculated by principal component analysis. We used this method to reduce the number of variables subjected to logistic regressions. The total number of insertions and the total copulation duration per female correlated positively (r = 0.882, N = 39, p<0.001). A principal component analysis run on these two variables extracted one factor with eigenvalue higher than one that explained 92% of the total variance. We termed this factor ‘copulation effort’ and used its regressed scores in further analyses.

Female mass was excluded from analyses because, probably due to missing values, the parameter covariance matrix could not be computed by the program.

In addition, we tested what factors influenced the occurrence of molting in adulthood. We used the number of males with which a female copulated (EG group), AP formation, egg-sac laying, copulation effort and weight as independent variables.

All statistical analyses were done using IBM SPSS Statistics 19. Reported *p*-values are two-tailed tests, with α = 0.05.

## Supporting Information

Video S1 ***Nephila pilipes*** **mating: male approach followed by unsuccessful insertion attempt.**
(MPG)

Video S2 ***Nephila pilipes*** **mating: male successful insertion.**
(MPG)

Video S3 ***Nephila pilipes*** **mating: male performing mate-binding in-between insertion attempts.**
(MPG)
